# Upregulation of the tight junction protein occludin: effects on ventilation-induced lung injury and mechanisms of action

**DOI:** 10.1186/1471-2466-14-94

**Published:** 2014-05-29

**Authors:** Mengjie Liu, Changping Gu, Yuelan Wang

**Affiliations:** 1 Department of Anesthesiology, Qianfo Mountain Hospital of Shandong University, Jinan 250014, China

**Keywords:** Ventilation-induced lung injury, Tight junction protein, Occludin, PKC inhibitor

## Abstract

**Background:**

Occludin, a tight junction protein, plays an important role in maintaining the integrity of the lung epithelial barrier; however, its role in ventilation-induced lung injury has not been explored. Here, we measured the expression of occludin with different tidal volumes. Our study indicated that the level of occludin was significantly decreased and alveolar permeability was increased owing to acute lung injury.

**Methods:**

Thirty healthy Wistar rats (15 female, 15 male) weighing 250–300 g, were randomly divided into 5 groups (n = 6 in each group): a control group (group C), a low tidal volume group (group L), a low tidal volume + protein kinase C(PKC) inhibitor group (group L + P), a high tidal volume group (group H) and a high tidal volume + PKC inhibitor group (group H + P). Tracheas of rats in the control group underwent incision without any special treatment. The other four groups were mechanically ventilated for 4 h. The rats in groups L + P and H + P were treated with a PKC inhibitor (bisindolylmaleimide I, 0.12 mg/kg) by intramuscular injection 1 h before anesthesia. Rats were sacrificed after mechanical ventilation. Specimens of lung tissues were harvested. Lung pathological changes were observed using an optical microscope, and lung wet/dry weight ratio was measured. The occludin protein level was assayed by immunohistochemistry and Western blotting.

**Results:**

HE staining and immunohistochemistry results showed that occludin was mainly located in alveolar epithelial cells and some alveolar endothelial cells. The lung injury and alveolar edema were more serious in high tidal volume groups than in low tidal volume groups. Occludin expression was reduced and PKC activation was increased in rats in the high tidal volume groups compared with rats in the low tidal volume groups. Rats that were pretreated with the PKC inhibitor had less pulmonary edema induced by the high tidal volume ventilation.

**Conclusion:**

Mechanical ventilation can activate the PKC signaling pathway and tight junction proteins participate in this pathway. Up-regulation of occludin can reduce ventilation-induced lung injury.

## Background

Ventilation-induced lung injury (VILI) is characterized by increased alveolar–capillary permeability, leading to an influx of protein-rich edema fluid and inflammatory cells into lung alveoli. The root cause for this is that the integrity of the alveolar membrane is damaged. Tight junction (TJ) proteins, including Zo-1, claudin and occludin [1], play an important role in maintaining the integrity of the lung epithelial barrier [2]. Previous studies have shown that intracellular protein kinases such as protein kinase C (PKC) and Src, and the protein phosphatases PP2A and PP1, when stimulated by inflammation, calcium or hydrogen peroxide, can phosphorylate or dephosphorylate occludin, leading to the degradation or synthesis of TJs [3]. However, the effect of PKC inhibitors on occludin protein expression has not been clearly demonstrated; this study aimed to investigate the effect of PKC inhibition on occludin protein expression in rat lung tissue, and the underlying mechanisms of occludin in VILI.

## Methods

### Materials

Thirty healthy Wistar rats (15 female, 15 male), weighing 250–300 g, were provided by the Laboratory Animal Center of Shandong Traditional Chinese Medicine University. All animal procedures were reviewed and approved by the Laboratory Animal Ethics Committee of Shandong University. The animal ventilator ALC-V8 was purchased from Shanghai Alcott Biotech Co., Ltd (Shanghai, China). Rabbit anti-occludin polyclonal antibody was purchased from Invitrogen. Rabbit anti-PKC α antibody was purchased from Beyotime Institute Biotechnology. The immunohistochemical staining kit was purchased from Beijing Zhongshan Biotechnology Company. The PKC inhibitor bisindolylmaleimide (BIM) was purchased from Cayman American.

### Grouping and processing

The rats were randomly divided into five groups (n = 6 in each group): a control group (group C), a low tidal volume (VT) group (group L), a low VT + PKC inhibitor group (group L + P), a high VT group (group H), a high VT + PKC inhibitor group (group H + P). Tracheas of rats in the control group underwent incision without mechanical ventilation. The other four groups were mechanically ventilated for 4 h [4,5]. The rats in groups L + P and H + P were pretreated with a PKC inhibitor (bisindolylmaleimide I) 0.12 mg/kg 1 h before anesthesia.

### Experiment protocol and samples harvesting

All rats were anesthetized with 10% chloral hydrate (3.5 ml/kg, i.p.) and kept in a supine position on a heating lamp to maintain animal temperature at 36–37°C. Subsequently, one 24-gauge cannula was inserted into the carotid artery drawing arterial blood samples. Each rat was intubated with a 16-gauge cannula and connected to the ALC-V8 animal ventilator for 4-h mechanical ventilation except for rats in group C. Ventilation parameters were set as follows [6]: ventilation with a high VT of 42 ml/kg, low VT of 7 ml/kg, a respiratory rate of 40 times/min, I/E ratio of 1:2, and a fraction of inspired oxygen (FiO_2_) of 21%.

At the end of the ventilation period, the rats were sacrificed by exsanguination, and the arterial blood was quickly harvested. The right lung upper lobe was quickly frozen in liquid nitrogen for Western blot analysis, and the remnant right lung tissue was fixed in 4% paraformaldehyde for 48–72 h for HE and immunohistochemical staining. The left lung was used to measure the wet lung weight; after that, the tissues were placed in an oven and maintained at a temperature of 70°C for 72 h to gain the final dry weight. Then, we calculated the pulmonary W/D ratio to quantify the magnitude of pulmonary edema.

### Immunohistochemical staining

Hematoxylin-eosin staining was performed to observe the pathological changes in these groups. The infiltration of neutrophils was observed under an optical microscope. Immunohistochemical staining was performed on 5-μm-thick sections after treatment with formalin. Sections were pretreated to promote antigen retrieval by steam in DIVA/citrate buffer (pH 7.0) solution, and quenched in 0.3% H_2_O_2_; then slides were incubated in 10% normal goat serum, followed by an anti-occludin antibody overnight at 4°C [1:80 dilution in phosphate-buffered saline (PBS)]. The next day, the slides were washed in PBS and incubated for 30 min with goat anti-rabbit secondary antibody. After being washed in PBS, the sections were developed with the chromogen diaminobenzidine tetrahydrochloride (DAB), and some of the sections were counterstained with hematoxylin.

### Western blot assay

The tissue fragments were lysed in radioimmunoprecipitation assay buffer supplemented with a cocktail of protease inhibitors, and then centrifuged. The lysate was collected and protein concentration was determined using a bicinchoninic acid protein assay kit (Beyotime). Equal amounts of protein were denatured and separated on 10% SDS-PAGE gels and then transferred to polyvinylidene difluoride membranes. The membranes were blocked with 5% skim milk and subsequently incubated with antibodies against occludin (1:250), PKC α (1:1000) and GAPDH (1:3000) overnight at 4°C. Then, the membranes were incubated with secondary antibody (1:5000). The membranes were then washed and the protein bands were detected using enhanced chemiluminescence substrate (Cwbiotech). Relative band densities of the various proteins were measured from scanned films using Image J Software.

### Statistical analysis

Statistical analysis was performed using the SPSS 17.0 statistics package. All data are expressed as the means ± SEM. One-way ANOVA with a post-hoc Tukey’s honestly significant difference test was used to determine the significance of differences among groups. P values of ≤ 0.05 were considered significant for rejection of the null hypothesis.

## Results

### High VT mediates the decrease in occludin levels and the activation of PKC kinase

**Figure 1:**
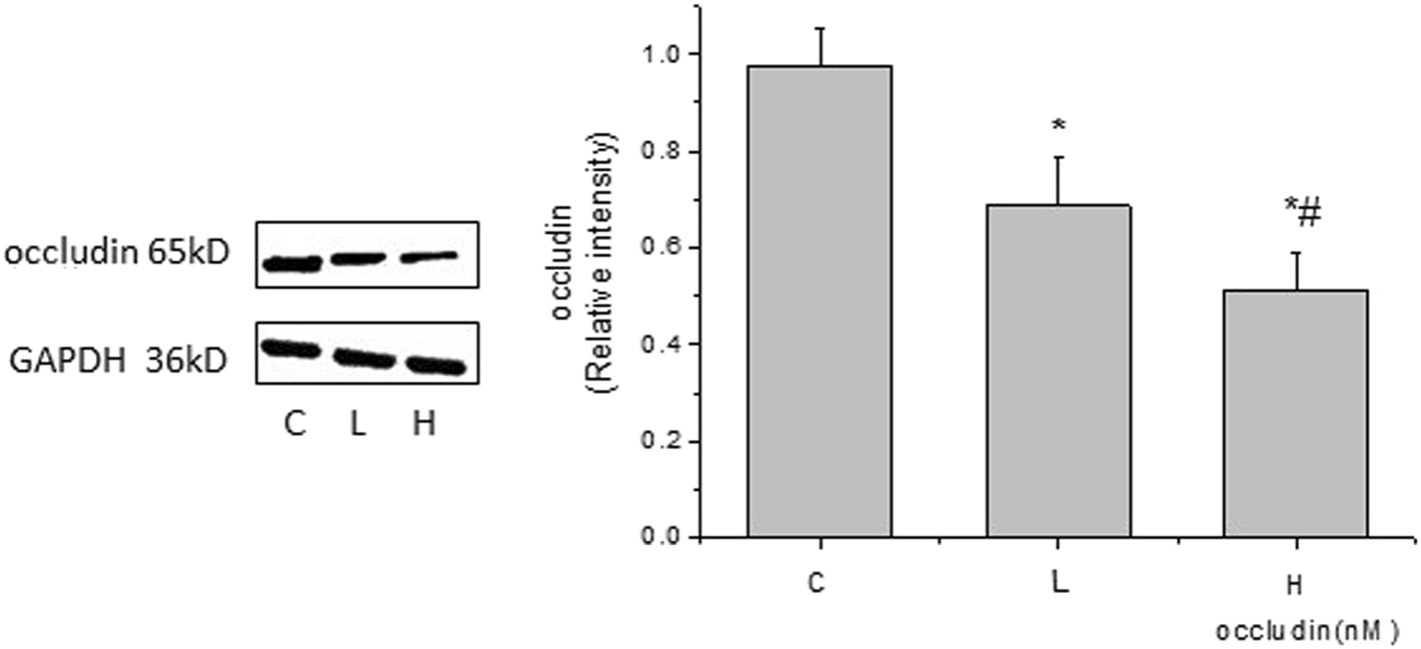
**Expression of occludin in different tidal volume ventilation groups (Western blotting).** (C) control group, (L) low tidal volume group, (H) high tidal volume group. The low tidal volume was 7 ml/kg; the high tidal volume was 42 ml/kg. The density of proteins in the control group was used as a standard to compare relative densities in the other groups. *P <0.05 vs. control. #P <0.05 vs. group L. Data are representative of three independent experiments.

**Figure 2:**
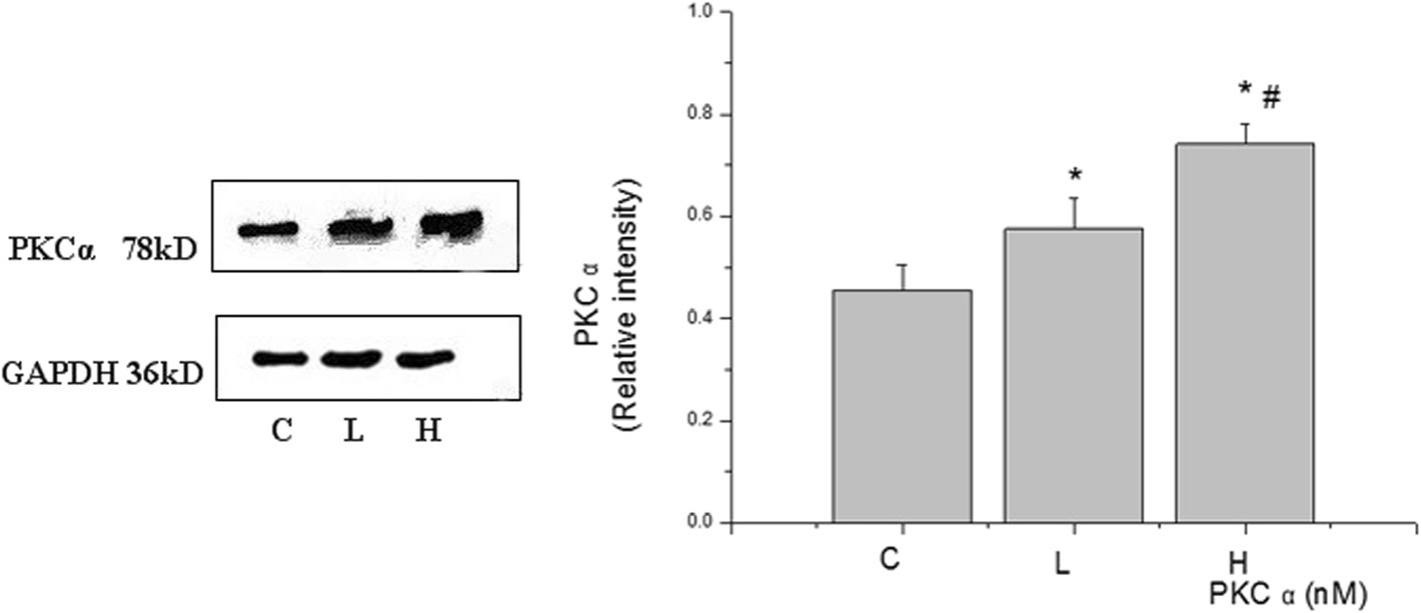
**Expression of PKC α in different tidal volume ventilation groups (Western blotting).** (C) control group, (L) low tidal volume group, (H) high tidal volume group. The low tidal volume was 7 ml/kg; the high tidal volume was 42 ml/kg. The density of proteins in the control group was used as a standard to compare relative densities in the other groups. *P <0.05 vs. control. #P <0.05 vs. group L. Data are representative of three independent experiments. Results are means ± standard error.

Rats were ventilated for 4 h; then, the changes in occludin levels and PKC activation were detected by Western blotting at different VT ventilations. Expression of occludin was significantly decreased in the high VT ventilation group (Figure 1). The activity of PKC in lung tissue was examined 4 h after VILI and the PKCα level was found to be increased in the high VT group compared with the level in the control group (Figure 2). However, the decrease in occludin level and the activation of PKC were not as obvious in the low VT group, being close to the levels observed in the control group, indicating that VILI could reduce the expression of occludin and activate PKC in a volume-dependent manner.

### PKC inhibition alleviates lung injury and pulmonary edema caused by mechanical ventilation

**Figure 3:**
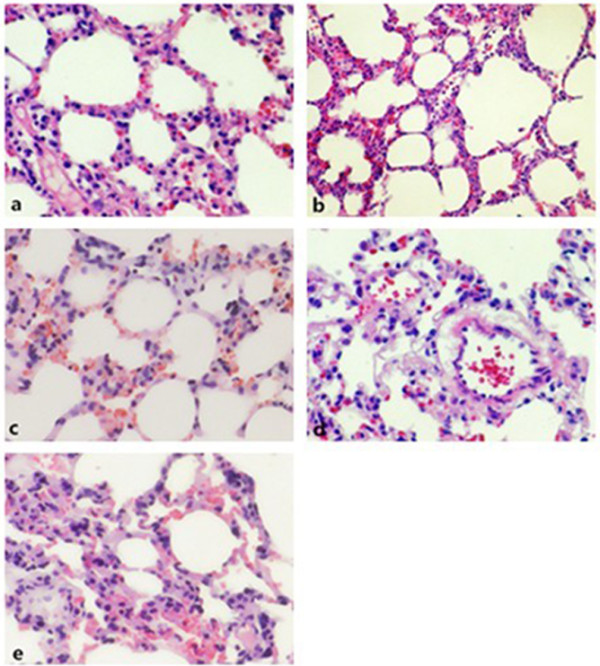
**Histological observation of lung injury in the control, L, L + P, H and H + P groups.** Lung tissue sections were stained with hematoxylin-eosin. Photomicrographs were taken at 400× magnification. One representative image for each of the lung microscopic photograph in the **(a)** the control group, **(b)** the low tidal volume group, **(c)** the low tidal volume + PKC inhibitors group, **(d)** the high tidal volume group, **(e)** the high tidal volume + PKC inhibitors group in three independent experiments is shown. The low tidal volume was 7 ml/kg; the high tidal volume was 42 ml/kg.

For the lung injury induced by ventilation, representative HE-stained sections of the lung tissues from control, L, L + P, H and H + P rats are presented in Figure 3a–e. The lung tissues of rats in the H group showed severe edema, alveolar hemorrhage, inflammatory cell infiltration and a destroyed pulmonary architecture. These findings were not obtained in the L group, and the pulmonary architecture was rarely affected with only mild edema and alveolar hemorrhage in the L + P and H + P groups.

Additionally, lung W/D ratio was calculated to assess pulmonary edema. As shown in Table 1, the lung W/D ratio increased slightly in the L group but significantly in the H group compared with the control group (5.85 ± 0.05 vs. 4.36 ± 0.11, p < 0.05). However, BIM pretreatment significantly reduced the degree of increase in lung W/D ratio in the L + P and H + P groups compared with the amounts in groups L and H (4.56 ± 0.06 vs. 5.25 ± 0.05, p < 0.05 and 4.89 ± 0.07 vs 5.85 ± 0.5, p < 0.05, respectively).

**Table 1:** **The ratio of Wet/Dry weight in rat lung** ($\left(\bar{x}\pm s\right)$)

**Groups**	**Ratio of wet/dry**
Group C	4.25±0.09
Group L	5.29±0.06^*^
Group L+P	4.61±0.05^*#^
Group H	5.89±0.06^*#^
Group H+P	4.91±0.08^*^&

Compared with group C, ^*^P<0.05; Compared with group L, ^#^*P<*0.01; Compared with group H, & *P<*0.05.

### Distribution of occludin and PKC activation and their relationship with the decrease in occludin level induced by mechanical ventilation

**Figure 4:**
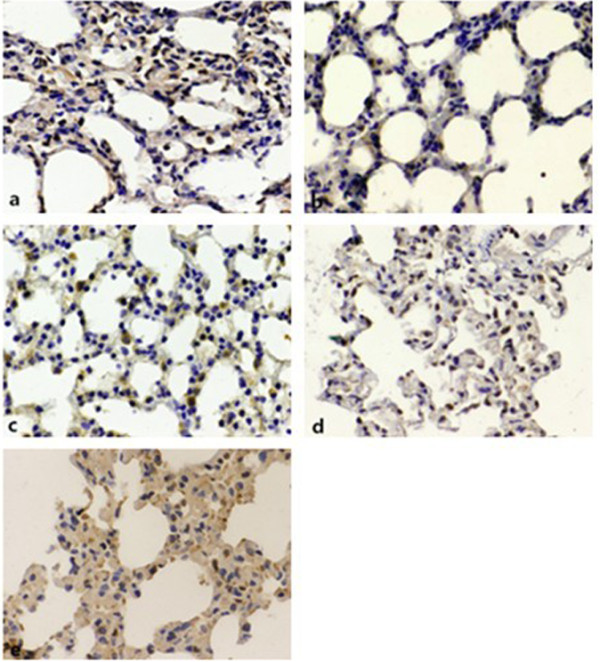
**Distribution and expression of occludin (immunohistochemistry, ×400).** Immunohistochemical staining for occludin protein in vascular endothelial cells and alveolar epithelial cells in paraffin sections of rat lungs 4 h after ventilation. **(a)** control group, **(b)** low tidal volume group, **(c)** low tidal volume + PKC inhibitors group, **(d)** high tidal volume group, **(e)** high tidal volume + PKC inhibitors group. The low tidal volume was 7 ml/kg; the high tidal volume was 42 ml/kg. Brown particles represent occludin-positive expression. Data are representative of three independent experiments.

**Figure 5:**
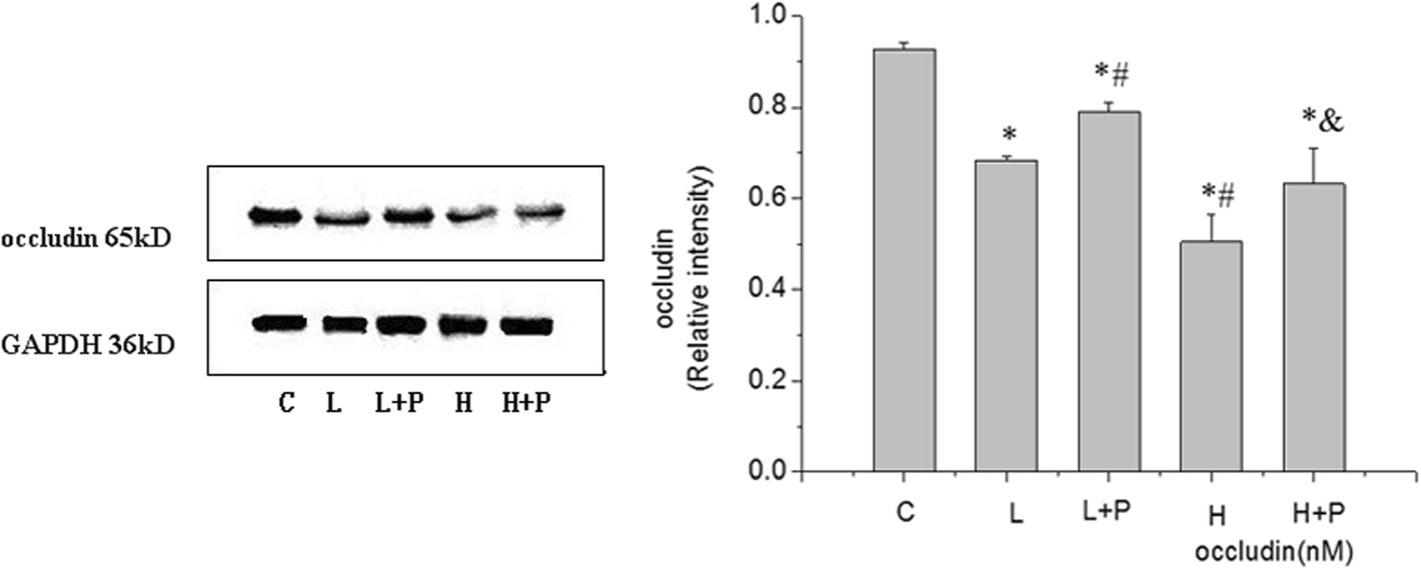
**Effects of ventilation and the PKC inhibitor BIM on occludin expression in rats.** Rats were subjected to the low tidal volume (7 ml/kg) or the high tidal volume (42 ml/kg) for 4 h. Groups L + P and H + P were pretreated with BIM 0.12 mg/kg 1 h before ventilation. After experiments, the rats were killed and occludin protein expression level was determined by Western blot analysis. The density of protein in the control group was used as a standard to compare relative densities in the other groups. *P <0.05 vs. control; #P <0.05 vs. group L; & p < 0.05 vs. group H. Data are representative of three independent experiments. A P value <0.05 indicates statistical significance.

The distribution of occludin in acute lung injury tissue was assessed using immunohistochemistry and Western blotting. Immunohistochemistry revealed that occludin was typically located in alveolar epithelial cells and vascular endothelial cells (Figure 4a). Researchers have shown that occludin is a substrate of PKC [3]. To confirm the activation of PKC and the reasons for ventilation-induced occludin degradation, we measured PKC levels and used a PKC inhibitor to pretreat the rats before ventilation for 4 h. Figures 4 and 5 show that the level of occludin decreased differently in each group compared with group C, and that the expression of occludin was significantly reduced in group H (Figure 4b–e). It was also found that PKC activation was upgraded and the PKC inhibitor prevented ventilation-induced occludin degradation. The content of occludin was higher in the rats in the pretreated groups than in those in the non-treated groups. Therefore, we considered that ventilation-induced lung injury and alveolar edema might be related to PKC activation and the degradation of occludin.

## Discussion

The main outcome of VILI is the formation of pulmonary edema, and the main cause of this is that the integrity of the alveolar membrane is damaged and permeability is changed [2,7]. In this study, we established a rat model of VILI with the aim of investigating the relationship between PKC activation and the decrease in occludin levels induced by ventilation. We used low VT (6–10 ml/kg) and high VT (35–42 ml/kg) in this study [4,8]. Therefore, we established a VILI model through setting high VT at 42 ml/kg ventilation for 4 h and with low VT of 7 ml/kg as a control. After the experiment, we found that the lung tissues of rats in the H group showed severe edema, alveolar hemorrhage and destroyed pulmonary architecture, which were not obvious in the L group. When the PKC inhibitor BIM was used to pretreat rats, the W/D ratio was lower than that in non-treated rats and the decrease in occludin expression was partly inhibited. Collectively, these findings may indicate that alveolar edema is associated with high VT and the decrease in occludin expression.

Adherens junctions (AJ) and TJs are important structures for maintaining the alveolar membrane barrier and permeability in pulmonary vascular endothelial cells and epithelial cells [9,10]. The relative impermeability of the alveolar epithelium to paracellular solute diffusion is predominantly regulated by TJs [11,12]. Occludin is a transmembrane protein of epithelial tight junctions [13]. The C-terminal domain of occludin interacts with ZO-1, ZO-2, and ZO-3; this interaction is required for the assembly of occludin into the tight junctions [11,14,15].

TJs are an important part of the lung alveolar epithelial barrier, and the function of occludin is closely related to the occurrence and development of many diseases [16]. There is evidence that occludin is related to gastro-intestinal diseases and cardiovascular diseases [17-19]. Studies have found that endotoxemia decreases pulmonary epithelial barrier function, activates NF-κB in lung tissue, increases pulmonary iNOS expression and decreases the expression of the TJ proteins occludin and ZO-1 [20]. New studies have found that the expression of occludin and ZO-1 is closely related to the destruction of the alveolar epithelial barrier [21]. This study aimed to explore the expression of occludin in rats with different tidal volumes. We found that the expression of occludin was affected in a volume-dependent manner. High VT ventilation reduced occludin protein expression significantly in rat lungs, while low VT just reduced occludin expression slightly (p < 0.05). Mechanical ventilation-induced acute pulmonary edema lung injury was caused by the reduction in occludin expression, degradation of tight junctions, decreased integrity of the alveolar membrane, and increased alveolar membrane permeability.

PKC is a generic term used to describe the largest serine/threonine directed kinase subfamily currently known [22]. Studies suggest that mechanical ventilation can lead to the elevation of intracellular Ca^2+^, then induce the activation of PKC, eventually activating c-fos causing lung injury [23]. Studies have shown that serine/threonine phosphorylation status plays an important role in regulating occludin^’^s function, and that PKC plays an important role in the phosphorylation of serine or threonine residues [14,24]. To date, 10 kinds of PKC subtypes have been identified in mammalian tissues; these are divided into three groups (A, B and C). Group A, known as classical or conventional PKC, consists of PKC α-, β1-, β2- and γ. PKC α can be considered a good representative of PKC molecules. Therefore, we examined its expression with different VTs. PKC was activated during high VT ventilation, while Western blotting revealed that the expression of PKC α increased with increasing tidal volume. It was slightly increased in group L and significantly increased in group H, while the expression of occludin reduced slightly in group L and significantly in group H. These findings indicate that there is a relationship between the activation of PKC and the loss of occludin, PKC activation inhibits the expression of occludin, so we pretreated rats with PKC inhibitors to explore the relationship between the expression of occludin and VILI.

Bisindolylmaleimide I (BIM) is a highly selective, cell-permeable, and reversible protein kinase C (PKC) inhibitor (Ki = 14 nM) [25]. The dose of BIM was determined according to the results of a previous study [26]. We found that the lung W/D ratio in the other four groups underwent different degrees of increase compared with group C (^*^p < 0.05), while the lung W/D ratio in rats in the groups pretreated with BIM was lower than in the non-treated rats (^#^p < 0.05, & p < 0.05). The expression level of occludin showed the opposite trend: Western blotting showed that, after pretreatment with BIM, the loss of occludin expression was partly inhibited (^#^p < 0.05, &p < 0.05) (Figure 5) and the acute lung injury was relieved. Considered together, these findings may indicate that occludin has an important role in maintaining the integrity of alveolar epithelia cell conjunction, and that alveolar edema induced by high VT is associated with the reduction in occludin expression owing to PKC activation.

## Conclusions

Mechanical ventilation can activate the PKC signaling pathway and induce a reduction in occludin expression in a volume-dependent manner. Pretreatment with a PKC inhibitor increased occludin expression, and could reduce or delay the mechanical VILI. This suggests that VILI may be associated with the reduction in occludin level, which may a result of PKC activation. The precise mechanism still needs to be further explored.

## Competing interests

The authors declare that they have no competing interests.

## Authors’ contributions

ML and CG carried out the animal experiment, participated in the molecular biology studies, ML carried out the immunoassays, participated in performed the statistical analysis and drafted the manuscript. YW participated in the design and conceived of the study, and participated in its design and coordination and helped to draft the manuscript. All authors read and approved the final manuscript.

## Pre-publication history

The pre-publication history for this paper can be accessed here:

http://www.biomedcentral.com/1471-2466/14/94/prepub
